# How Inebriety Might Be Prevented by Early Education

**Published:** 1910-10

**Authors:** Tom A. Williams

**Affiliations:** M. B., C. M., Edin., Washington, D. C.


For Texas Medical Journal.

### How Inebriety Might Be Prevented By Early Education.

BY TOM A. WILLIAMS, M. B., C. M., EDIN., WASHINGTON, D. C.

At a recent convention of the National Society for the Study of Alcohol and Other Drug Habits,[1] not the least interesting feature was the consideration of the psychological bearings of habitual drug-taking. That drug-taking is a habit, needs no argument; but habits have psychological origins; and it is only by studying these that we shall learn to prevent the formation of the habit.[2]

Now, the desire for stimuli, of which drugs are only one, proceeds from a psychological state of dissatisfaction, of discontent,[3] or, in a lesser degree, of vague discomfort, ennui, hankering. To terminate this feeling, a longing ensues generally for some definite sensation or action, sometimes for nothing in particular except excitement, in whatever form it be. Some extraordinary means have been adopted by patients to satisfy this longing; e. g., one of Janet's psychasthenics would slowly drop boiling water upon her naked feet when the state of longing came over her.[4] The scalding was more tolerable than "the blues." A very common reaction against the state of vague discontent felt by these patients is the *fugue,* which the French writers have described so fully. The wandering Jew, the medieval type of this affliction, still exists today[5] on the continent of Europe; and in this country the same tendency to escape from oneself and immediate surroundings is the basis of the mode of life of many a tramp and hobo. Sexual perversions, also, often originate in this way. Vague discontent with oneself or with the world around one much more often than a real active desire for betterment, is the spring from which arises most of the mysticism of our own day, whether this is clothed in the form of the so-called saintly absorption, which was so common in medieval times or whether it takes the form, as is often the case nowadays, of adherence to a cult such as New Thought, Yogiism, Christian Science, or even reliance upon nebulously conceived hypnotism, mental suggestion and what not. The extent of the vogue these movements have acquired is due to their response to a very real need. The fact that they obtain this following, even though clothed in such absurdities, is due to the ignorance of the laity about the laws of their own minds, and also to the modicum of truth which the doctrines of each contains. But they all tend to perpetuate the incomplete conception, ignorance of the real man, and dependence upon the will of others, and the support of a figment of the imagination. In saying this, I am not endeavoring to belittle the real service they are doing to many a weary-minded mortal, but to point out that the modicum of good they accomplish is accompanied by much harm, and that their doctrines are a hindrance to the ultimate progress of humanity towards self-realization and power over itself and its environment. The records of any insane asylum, and, indeed, my own case-books, afford adequate proof of this. A considerable percentage of a neurologist's patients suffer from no organic disease, but are in need of being shown how to manage their nervous life, and more particularly their emotions. And these are the people who, if by a happy chance they do not find the consulting room of some one who understands them, end in drink, suicide, or the asylum. It was for such individuals that the medieval church provided "the retreat," during a sojourn in which the perturbed mind might find solatium for its distress, freedom from its too exacting environment, repose for its intellect, and decision in place of its hesitancy and doubt, and with it all a quiet though fervid orientation towards an inspiring hope.

This is the state of mind with which we have to deal in the unfortunate who is addicted to drugs; but the means of doing so can not be touched upon without unduly prolonging this discussion. It is to its prevention I wish to confine myself. What, then, is its cause? We are learning this by our studies of genetic psychology,[6] of the child as it grows by seeing a trait in its first simple appearance in the infant and tracing it to full maturity in the adult complexity.

The mother who seeks out every caprice of her child to satisfy it, is laying the train for future explosions of uncontrolled impulse. The mother who neglects her child to the point of compelling him to seek amusement at all costs from any passer-by, and hence to discard everything which does not immediately please, is incurring many chances of her boy developing a habit of immediate satisfaction at all costs. Again, the parent who allows doctrinaire rigidity to alienate him from the sympathetic understanding of his child's innocent and harmless turbulence is driving him to seek elsewhere the modicum of solace which at least every child at times requires. A frequent outcome of this is the alternation of stoical self-suppression and outbursts of indulgence in what is believed to be wrong.

Whether the indulgencies of states of feeling find their accentuation in alcohol, or whether they use some other aid, is a mere accident of environment. This accidental nature of the response to longing is shown by the experiments of Pawlow[7] with dogs. Thus, by association of ideas, ringing of a bell could determine gastric flow, which could be again inhibited by the showing of a whip; and in turn any impression could be substituted for these and produce pleasurable or painful emotions as well as increase or decrease in the secretions. In another case of Féré,[8] the attempt to force out of the house a dog suffering from agorophobia caused such terror that the evacuations escaped involuntarily.

In general, a habit-reflex forms, and the early indulgencies are those which persist; but it must be remembered how much greater is a desire for spiritual sustenance and comfort when the stress of independent industrial life combines with the decline of youthfulness. Hence, the pathological indulgence of feeling in hurtful acts may be postponed quite late although the pathological feelings had hitherto been there, though restrained by self-respect, religion, the sake of decency, or fear of the criminal law.

Psychological experiment shows how persons differ from day to day in mental capacity. Physiological experiments exhibit the difference in bodily secretions and inactivity. Such oscillations are as true of the feelings, depending, as these do, upon bodily changes and mental impression. Nearly all of us, then, must necessarily encounter phases during which our feeling is one of incapacity, even of inaptitude, discontent, dislike of our surroundings, anxiety, etc. To support these unpleasant states, a certain fortitude is required, unless one chooses to put an end to the state of feeling by some stimulus. The outcome of this course is the need for a very large stimulus to do away with a quite trifling feeling, for the power of resistance progressively decreases by non-use, especially when a ready satisfaction is within reach. The-immediate-satisfaction-of-desire-at-all-cost is a habit which can be made or unmade at the will of the educator, and it is towards this factor that the prevention of inebriety must be directed. Even persons emotionally unstable may be readily taught to provide against the extra load this might mean. In this respect, the ancient religions showed themselves empirically more efficacious; for the reinforcing effect of active movement upon our thought is now an established fact. Will is nothing more than the balance of the concomitant stresses towards movement, and pedagogy has taught us that present methods lack woefully that dymogeny without which education is a mere name. In this respect, the modern world has been injuriously dominated by the arm-chair philosophers, who have neglected the facts of life, and, above all, the genetic factor. The notions of experimental science have not yet sufficiently penetrated the teaching of ethics. This has been left entirely in the hands of persons whose point of view is hopelessly vitiated by the artificialities of outworn conceptions of the universe and of the mind of man, which are maintained by the traditions of popular literature, academic philosophy and ecclesiastical dogmata and ritual whose nature precludes adjustment.

Moods and emotions, as Spencer[9] long ago showed, are the determinants of conduct. The direct power of idea and reason in modifying behavior has strict limits. However, the indirect effect is tremendous. Prevision, however, is the essential element of this control, and this prevision must occur before the formation of emotional habit. It is the very early years which form these habits. The perversion of infancy and childhood through the neglect by parents of the knowledge we have for guiding the disposition of a child is most reprehensible. The overthrow of the method of obedience to the arbitrary desires of a parent ignorant of the evolution of the child's mind and has been followed by the equally obnoxious "laissez faire" methods, conspicuously shown in the United States, where emotions and behavior at least are concerned. The abolition of obedience as such has enthroned the immediate impulse as the ruling factor.

Unless education in ethics becomes as kinetic when applied to morality as it now is with regard to business and the law, it will continue sterile. To do this, we must order the consequences of our children's act in conformity with their powers of observation and inference. Where manners and morals are concerned, people act indiscriminately, conventionally, impusively or indifferently; thanks to the apology for training they have received.

Again, the constant attempt to arrest the mental activity of the child by thwarting even his healthy impulses deprives him of initiative, and he becomes discontented, unless entertained by others. This want of resourcefulness is a sure forerunner of ennui, of the loafing habit. To prevent this, method, as in the universities, is more important than results. Didactically memorized precepts have no meaning to the childish intelligence; whereas, education by deeds is pregnant with results. The events upon which the child has to base his inductions must be carefully chosen by the parent to conform to the limits of his intelligence, and, of course, must not be at variance with natural law; for example, when he shows cruelty to an animal, there is no real efficacy in telling him he is a naughty boy, but a great deal in presenting him with a pet able to resent and produce discomfort. Again, if he shows fear of an animal, exhortation meets no stored memories upon which to bear, but the familiarity gained by fondling an animal which does not hurt soon substitutes a new emotional for that of fear.

The first, intermediate and last art, that of living in relation to others, is taught only in the most haphazard or arbitrary way or entirely neglected. Is it not reasonable, therefore, to demand for this at least the beginning of a graded curriculum, in which examples must be worked out by the student and in which he is taught "rule" by "practice"? The ethics which is taught in the rule-of-thumb way of the average family is still that of rudimentary survivals. It is conspicuous for its poverty in such criteria of modern civilization as justice, liberty, courtesy, altruistic sympathy. The natural good impulses of the child are even artificially checked and twisted, his reasoning from cause to effect, where conduct is concerned, is neglected or obstructed; he is thus confused, and finally discouraged into sadness or indifference, and is bred into a despondent or happy-go-lucky man, ethically speaking. Even if knowledge and freedom are ultimately attained, it remains difficult to throw off the affective accompaniments of conduct first practiced under such brutish auspices.[10]

The responsibility for the different attitudes which the child observes in his parents towards moral questions as against others must be laid to the door of religion; for the sacrosanct connotations of supernaturalism, which pervaded morality in days of ignorance and repression, have still survived, on account of the want of its scientific study and practice. On the one hand, we find a perpetuation into adult mental life of the helplessness and irrationality of the child; and at the other extreme is taught the inherent damnableness of human nature unless justified by faith. Need one insist upon the effect of either of these attitudes upon the cultivation of the power of observation, inference and of reasoning in general?

Its effect upon the sentiments has been even worse; for, in the child of careless or indifferent mind, these qualities have been perpetuated by the attenuation of their results into a state of happy expectancy that the Lord will take care of His own. The second extreme will fall most heavily upon the child who is inclined towards overconscientiousness. The neurologist almost daily is presented with examples where this morbid trend has been cultivated to excess by the religious atmosphere legated by the apostle of Geneva.

Now the cultivation of either the happy-go-lucky disposition or that of hyperconscientiousness is bad for that intellectual and affective poise which is the best safeguard against the psychological state favoring inebriety. A disposition towards carelessness is fortified by the constant leaning upon others; the scrupulous disposition is fostered by misplaced reliance upon so-called intellectual determinants of conduct. To the child, these are meaningless, because they are mere symbols of something he can not understand owing to want of motor experience. That which makes a concept effective is its motor element; without this it is quite incomplete. It might nearly be said that an idea which has never been kinetic is impossible; that indeed the notion is not in consciousness; all that is there is the simulacrum constituted by the verbal image. A familiar example is the child's "chart in heaven," which shows how little he was conscious of the real meaning of the Lord's prayer.

The truth of this is implied in the old provert, "example is better than precept," but the implication depends upon the fact that example can be understood, and hence rendered kinetic by imitation, while precept conveys comparatively small meaning.

These experiences must not be forced at undue age, or the painfulness of their acquisition will bring disgust instead of pleasure. As accomplishment is learned, the kinesthetic element tends to fall more and more into the background, and to be represented visually and auditorily; but it is, nevertheless, present, and once more emerges during states of mental dissolution. It is the real basis of knowledge, and the neural stresses entailed by its inhibition from activity have important functions in the associational processes. Examples abound.

Isaac Newton was at the foot of his grade at 12. He showed neither ability nor industry. Charles Darwin was not at all a studious bod. He writes, "To my deep mortification, my father once said to me, 'You care for nothing but shooting, dogs, and rat-catching.'" Rosa Bonheur, in her eleventh year, generally contrived to avoid the school-room, and spent most of her time in the woods. When placed with a seamstress, in order to learn to sew, she implored her father to take her away, which he did, and much perplexed left her entirely to herself, and Rosa, full of unacknowledged remorse for her incapacity and uselessness, sought refuge from her uncomfortable thoughts in his studio, where she learned her art as a solace in play. A vast majority of parents and teachers do not appreciate the tremendous possibility of character-building through play, and they try to subdue it in the child, thinking it is something he should overcome, forgetting that, when the time comes, it will pass out of his life; and will do so as naturally and readily as the tail of the tadpole is absorbed when there is need of the legs of the frog. The hilarious enthusiasm of childhood and youth will in time develop into the eager earnestness of the business man, the soldier, etc. As said Stanley Hall, "There is a sense in which all good conduct and morality may be defined as right muscle habits." As these grow weak and flabby, the chasm between knowing the right and doing it yawns wide and deep. As F. P. Robertson said, "Doing is the best organ of knowing." This must become the dominant note in the pulpit itself as soon as the preacher seeks to know what the soul really is.

That this is being realized is shown by the playgrounds movement, which in Germany is used as a developer of the invention and creative instincts, and for the growth of muscle, mind and morals. In England, this is done in the national games, which are a part of the curriculum in the better secondary schools. In these games, the masters themselves not only supervise, but participate, and in this way encourage fortitude and the spirit of fair play, and restrain, or at least guide, the exuberance and natural brutality of the boy. As a matter of fact, phylogeny shows us that the most valuable lessons of life should be learned in play.

But educators, unfortunately, think that they have discovered a better way than the natural one; and our little children were and still are forced against all instincts of life away from their play into schools, where, in many cases, play is rarely permitted. As a result, they are suffering from arrested development of the will, as well as of the emotions and the intellect. No wonder Froebel insisted, "Wouldst thou lead the child in this matter, observe him. He will show thee what to do." The child in a palatial nursery may lead a life even less desirable than that of those in shops and factories. He, too, may miss the stages of differentiation only possible with constant reactions to healthy environment. Even though not stunted physically, he is certain to be so mentally and morally; for, as James has said, "The boy who lives alone at the age of games and sports will usually shrink in later life from the effort of undertaking that which in youth would have been a delight, and so with traits of character, they must become reflexes in childhood and youth, or the opportunity for their development will have passed." Otherwise, we shall crush out characteristics upon which future strength depends and force the growth of untimely virtues, which will never become mature. Take pugnacity, for instance; it is generally suppressed in modern education, which forgets that the good man is not the man who never fights, but rather the one who does so and fights for the right and in defense of the downtrodden. Similar arguments may be used with regard to selfishness, anger, cruelty, rude humor, venturesomeness and other so-called evils. As a matter of fact, the boy who can not play, if he has had the opportunity, is not capable of work; for both work and play are merely the use of the surplus of energy after breathing, digestion, and circulation of the blood have been accomplished.

The superiority of play as against work in the development of a child's character is due to the interest it gives. This stimulates effort, without which development will be imperfect. Indeed, activity made without effort conduces to bad habits of action, slovenliness, and lack of will-power—the want of forcefulness.

Regarding altruism, play is again the best developer. The small child can not but be selfish; he can not see the need of co-operation. Group games will gradually teach this, for instance, little boys have no acknowledged captain; but, later, the efforts to play well and for the team to win, makes necessary the subordination of certain individuals for the good of the whole; and so, first a temporary, and, later, a permanent, captain must be selected. From this, develops a respect for law and order, and the will to submit to discipline, and amenability to the results of its infraction. The unselfishness thus derived is an active force in the future man's life; it is kinetic. Hence, we can no longer say that knowledge alone is power; and we may say again with Froebel, "A comparison of the relative gains through play of the mental and physical powers would scarcely yield the palm to the body: justice is taught, and moderation; self-control, truthfulness, loyalty, brotherly love, courage, perseverance, prudence, together with the severe elimination of indolent indulgence."

Premature attention to the inhibition of motor activities in the development of man prevents the development of the psychological systems, without which capacity can not be attained. Resolution becomes permanently "sickled o'er with the pale cast of thought"; and, moreover, not only are the activities incomplete, but those which develop are incommoded by the constant fear brought by an over-active conscience. As James has asked, "How can social intercourse occur in the sea of responsibilities and inhibitions, due to the self-centered horror of saying something too trivial and odious or insincere or unworthy of the company or inadequate to the occasion?"

Now the tremendous friction of a life of restraint upon normal activities causes nervous exhaustion; and this feeling is so painful that one really flies to what removes it. Hence, inebriety.

On the other hand, there is danger in the noncultivation of inhibition; for impulsiveness then rules; and this meets with innumerable inducements to intemperance of all kinds. But its cultivation must not conflict with ontogeny, and, above all, must be kinetic.

It is from these two extreme types that are mainly recruited the intemperate.

Hence, it is upon the study of morbid psychology that each and all must found their procedures, if they wish to build rather on rock than on sand and to hew a step more in the advance of humanity towards the perfection it seeks.

The remedy is the teaching of mothers to form healthy emotional habits in their children. The happy-go-lucky absolutism which so often asserts itself as capacity is sadly defective as such a guide for hesitating childhood. The mind, the emotions, and their management into a morality constitute the most difficult study and art. Woman's sphere is here, and is indeed a noble one; but instinctive motherhood has had its day. The women who aspire to bring up leaders of men in a nation which aims at future greatness must cease striving for vain things, and no longer confine their attention to superficialities; but do as their grandmothers did, and buckle, too, modestly, thoroughly to an understanding of that fascinating complexity—the heart and mind of the child.

REFERENCES.

1. The Alcohol Question in the United States. *Lancet.* London. (To appear.)

2. Williams—The Psychological Bases of Inebriety. *New York Medical Journal*, April, 1909.

3. Ragmont et Janet—Les Obsessions et la Psychasthenie. Paris, 1903.

4. Janet—Les Oscillations du Niveau Mental. Congrès de Psy., Rome, 1904.

5. Meige—Le Juif Errant a la Salpetrière. Paris, 1895.

6. Stanley Hall—Adolescence. New York, 1904.

7. Pawlow—Huxley Lecture. *Brit. Med. Jour.*, 1906.

8. Féré—Revu d'Hypnotism. 1892.

9. Spencer—Principles of Psychology.

10. Friedman and Gerlich—Studies on Paranoia. Trans. in Nervous and Mental Monograph Series. Lancaster, Pa., 1908.

See, also:

Leuba—*Amer. Jour. Religious Psychology*, 1906.

O'Shea—Dynamic Factors in Education, 1906.

11. Williams—Considerations as to the Nature of Hysteria. Inter. Clin., 1908.

12. Williams—Diff. Diag. of Neurasthenia and Some Affections of the Nervous System for which it is Often Mistaken. Archives of Diagnosis, January, 1909.

Dupré—La Mythomanie. Paris, 1906.

Ahumada (Thèresa de)—Le Chateau Interieur. (French Trans.)

Spencer—Education—Physical, Intellectual and Moral.

Gilman—Concerning Children.

Archibald—The Power of Play in Child Culture.

Swift—Mind in the Making.

---

There is often a "clear interval" after a stab wound of the heart, similar to that following laceration of the middle meningeal artery. —*American Journal of Surgery.*

